# Labial adhesion in a postmenopausal female: A case report

**DOI:** 10.1097/MD.0000000000020803

**Published:** 2020-06-26

**Authors:** Chun-Yo Laih, Chi-Ping Huang, Eric Chieh-Lung Chou

**Affiliations:** a Department of Urology, China Medical University Hospital; b School of Medicine, China Medical University, Taichung, Taiwan.

**Keywords:** case report, labial adhesion, postmenopausal female

## Abstract

**Rationale:**

Voiding difficulty is more common in males, although it is not uncommon in females. Female voiding difficulty can be caused by iatrogenic, anatomic, and neurogenic factors, and specifically urethra stricture, impaired detrusor contractility, primary bladder neck obstruction, and detrusor-external sphincter dyssynergia. Labial adhesion is a rare cause of female voiding difficulty.

The incidence of labial fusion has been reported to be 0.6% to 1.4% in children; however, the incidence in the elderly has yet to be fully elucidated.

**Patient concerns:**

We present the case of a postmenopausal and sexually inactive 76-year-old female patient who had nearly total vaginal and urethral occlusion due to labial adhesion. She had no underlying diseases and came to our clinic with a 10-month history of voiding difficulty, postmicturition dribbling, and involuntary urinary leakage when getting up.

**Diagnosis:**

A genital examination revealed nearly total fusion of the labia minor with only a 3-mm pinhole opening at the posterior end.

**Interventions:**

Treatment included surgical separation, the local application of estrogen cream, and self-dilatation. She also received an antimuscarinic agent to treat overactive bladder secondary to bladder outlet obstruction which was caused by labial adhesion.

**Outcomes:**

No surgical complications occurred. Moreover, no labial adhesion or voiding dysfunction was found immediately after the surgery or after 6 months of follow-up.

**Lessons subsections:**

Genital examinations are a basic but very important noninvasive skill for physicians. This case report highlights that genital examinations should be a priority for patients with gynecological or urological symptoms.

## 1 Introduction

Voiding difficulty is more common in males, although it is not uncommon in females. Labial adhesion is a rare cause of female voiding difficulty that is more commonly seen in elderly females. The incidence of labial fusion has been reported to be 0.6% to 1.4% in children; however, the incidence in the elderly has yet to be fully elucidated.^[1]^ In addition, no previous studies have reported the diagnostic tools, interventions, or outcomes of labial adhesion. Labial adhesion, which is also called synechiae of the vulva, vulvar fusion, agglutination or conglutination of the labia minora, and adhesion of the labia minor, is defined as when the labia minora or majora undergoes partial or complete adherence.^[2,3]^ The etiology is thought to be chronic inflammation or irritation of the vulval area, and a lack of estrogen and constant friction during mobility which leads to superficial skin epithelium denudation and slow progressive fusion of the labia.^[4–7]^ Labial adhesion is uncommon in elderly women, and they may be embarrassed to mention the condition when they see a physician. Therefore, the patients may have various symptoms, including voiding difficulty, urinary incontinence, and postmicturition dribbling.

## 2 Case presentation

A 76-year-old woman with no underlying diseases came to our clinic with a 10-month history of voiding difficulty, postmicturition dribbling, and involuntary urinary leakage when getting up. Her menstruation started when she was 14 years old, and the interval between menstrual cycles was prolonged to 1 time per 3 to 4 months and even to 1 time per year after 25 years of age. She had not had sexual intercourse since 40 years of age. No vaginal infections were noted except for 1 episode when she was 45 years old. No other symptoms were noted including fever, hematuria, flank pain, or pelvic pain.

**Figure 1:**
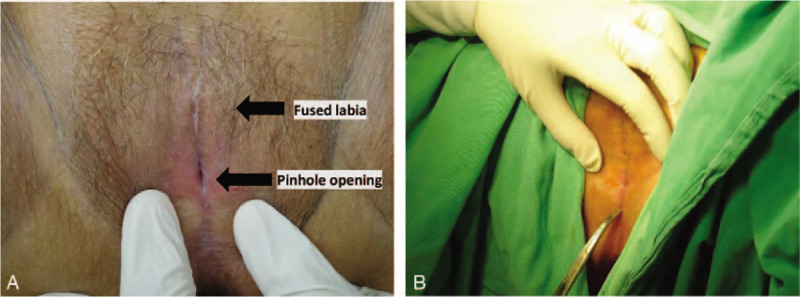
A and B, Near-total fusion of the labia minor with only a 3-mm pinhole opening at the posterior end.

Urine analysis was normal with no pyuria or hematuria. We then performed a genital examination, which revealed nearly total fusion of the labia minor with only a 3-mm pinhole opening at the posterior end (Fig. 1A and B).

**Figure 2:**
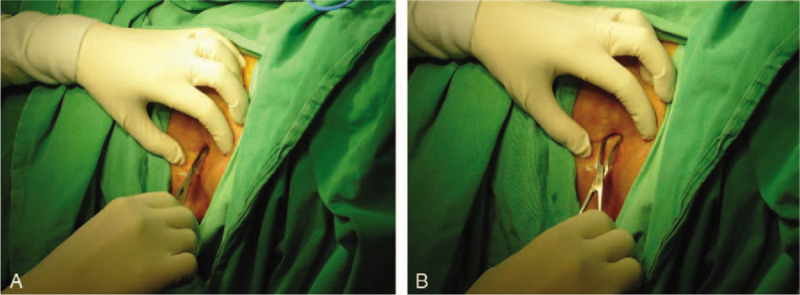
A and B, Blunt labial dissection and separation were done with Kelly forceps.

**Figure 3:**
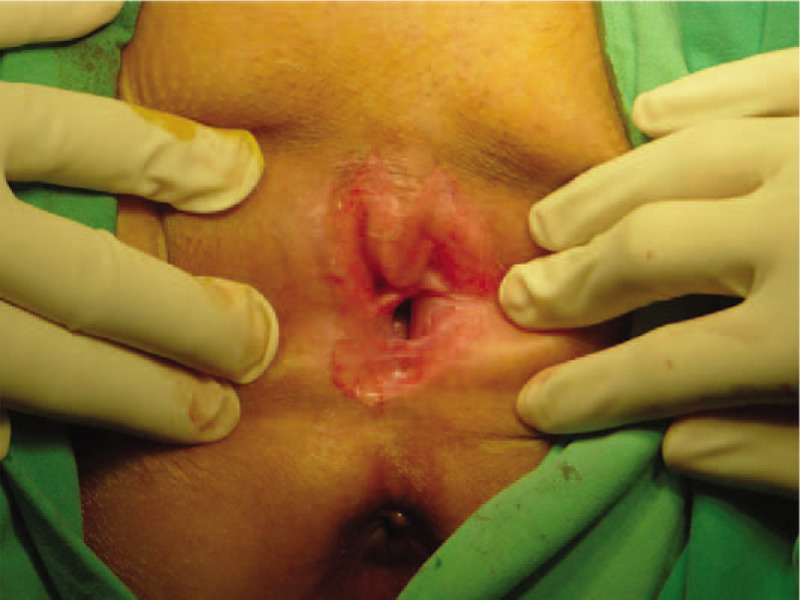
The labia minor was separated after the blunt dissection.

**Figure 4:**
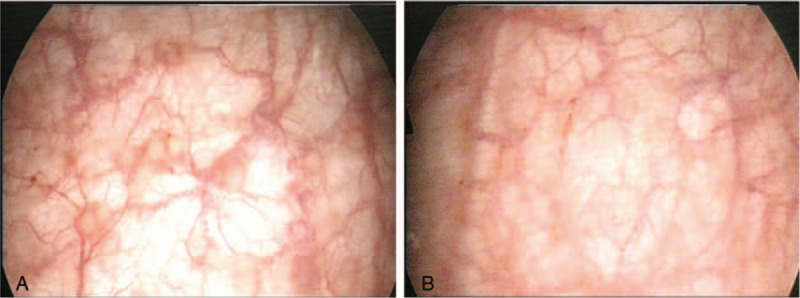
A and B, Cystoscopy showed cystitis cystica and mild trabeculation.

Surgical separation was arranged under spinal anesthesia, during which we used Kelly forceps to perform blunt dissection (Figs. 2A, B and 3). In addition, mild trabeculation and erythematous mucosal changes on the posterior bladder wall were found on cystoscopy (Fig. 4A and B). Cold cup biopsy revealed cystitis cystica. She was discharged 2 days after the surgery, and instructed to apply estrogen cream locally.

She returned to our clinic 1 month later, when we performed finger separation and taught her how to self-dilatate because of reocclusion. After 6 months of follow-up, no labial adhesion or urinary symptoms were noted.

## 3 Discussion

The exact incidence of labial fusion is unknown. In 1993, Norbeck et al^[1]^ reported an incidence rate of 0.6% to 1.4% in children. However, a review of the literature did not reveal any studies on the incidence of labial adhesion in the elderly.

The etiology of labial adhesion is unclear, although vulvovaginitis and mechanical irritation have been implicated as causative risk factors.^[8]^ In addition, chronic inflammation is thought to cause denudation of surface epithelium, thereby allowing the labia to adhere to each other and result in the obstruction of the introitus.^[9]^ A possible explanation for this condition is believed to be due to a hypoestrogenic state. However, if a hypoestrogenic state is the only explanation, labial agglutination would be expected to be more prevalent in postmenopausal elderly females.

The exact prevalence of labial adhesion in postmenopausal elderly females is unknown, and only a few case reports have documented this condition in postmenopausal women.^[9]^ In our case, oligomenorrhea may have been due to a hypoestrogenic state; however, vulvovaginitis or other inflammatory symptoms were not seen.

Labial adhesion may be asymptomatic or symptomatic according to the severity of adhesion. Symptomatic labial fusion often presents with vulval pruritus, and rarely with urinary incontinence, voiding difficulty, urinary retention, and dysuria.^[9,10]^ According to the case series of labial adhesion in 5 postmenopausal elderly women reported by Pulvino et al in 2008, 4 cases reported changes in urinary stream during micturition, with 1 describing the stream as “spray like” and 3 reporting “postvoid dribbling,” which is compatible with our case.

Physicians can detect any abnormalities of the external genitalia through careful genital examinations, and such examinations are the most simple and important diagnostic tool for labial adhesion. We performed a genital examination which revealed nearly total fusion of the labia minor with only a 3-mm pinhole opening at the posterior end (Fig. 1 A and B). The degree of labial adhesion varies according to the residual length of the introitus. No universal classification systems of labial adhesion upon genital examination were found in a review of the literature.

Standard treatment for patients with labial fusion includes topical estrogen cream, manual separation, or surgery. However, the most common treatment for cases of adult labial adhesion is adhesiolysis. For adult patients, estrogen therapy is not always successful as a first-line treatment, and surgery may be necessary.

A retrospective review by Mayoglou et al in 2009 revealed a rate of surgery after estrogen therapy failure of 27.4%, with a recurrence rate of 15.8%. The related symptoms included urinary tract infections, postvaginal dripping, vaginitis, and urinary frequency. However, this study only focused on prepubertal girls, and no data were reported for elderly females. In the case series reported by Bradford and Fischer, 7 patients with severe introital stenosis underwent surgical treatment. After surgery, none of the patients had total readhesion; however, 2 patients had partial refusion because of severe lichen planus and noncompliance.^[11,12]^ A review of the literature revealed no treatment guidelines for labial adhesion in postmenopausal females, probably due to the rarity of the condition.

## 4 Conclusion

Genital examinations are a basic but very important noninvasive skill for physicians, especially for urologists and family medicine doctors. However, elderly women may not touch their external genitalia, especially Asian women. In addition, due to a heavy clinical load and advances in modern medical knowledge and technology, laboratory and imaging studies are often arranged prior to a genital examination. This case report highlights that genital examinations should be a priority for patients with gynecological or urological symptoms.

## Author contributions

**Conceptualization:** Chun-Yo Laih, Chi-Ping Huang, and Eric Chieh-Lung Chou.

**Writing – original draft:** Chun-Yo Laih.

**Writing – review & editing:** Chun-Yo Laih, and Eric Chieh-Lung Chou.
